# Supplementary material for: A fault-intrusion-tolerant system and deadline-aware algorithm for scheduling scientific workflow in the cloud
Source: PeerJ Comput Sci. 2021 Nov 2;7:e747. doi: 10.7717/peerj-cs.747 (PMC8576554; doi:10.7717/peerj-cs.747)
Supplement: Supplemental Information 6 [file peerj-cs-07-747-s006.zip › Myproject1.1/docs/org/cloudbus/cloudsim/provisioners/BwProvisioner.html]

BwProvisioner


JavaScript is disabled on your browser.


- Overview
- Package
- Class
- Use
- Tree
- Deprecated
- Index
- Help

- Prev Class
- Next Class

- Frames
- No Frames

- All Classes

- Summary:
- Nested |
- Field |
- Constr |
- Method

- Detail:
- Field |
- Constr |
- Method


org.cloudbus.cloudsim.provisioners

## Class BwProvisioner

- java.lang.Object
- - org.cloudbus.cloudsim.provisioners.BwProvisioner

- Direct Known Subclasses:
  :   BwProvisionerSimple

  ---

    

  ```
  public abstract class BwProvisioner
  extends java.lang.Object
  ```

  BwProvisioner is an abstract class that represents the provisioning policy of bandwidth to
  virtual machines inside a Host. When extending this class, care must be taken to guarantee that
  the field availableBw will always contain the amount of free bandwidth available for future
  allocations.

  Since:
  :   CloudSim Toolkit 1.0

- - ### Constructor Summary

    Constructors

    Constructor and Description

    `BwProvisioner(long bw)`

    Creates the new BwProvisioner.
  - ### Method Summary

    Methods

    | Modifier and Type | Method and Description |
    | --- | --- |
    | `abstract boolean` | `allocateBwForVm(Vm vm, long bw)` Allocates BW for a given VM. |
    | `void` | `deallocateBwForAllVms()` Releases BW used by a all VMs. |
    | `abstract void` | `deallocateBwForVm(Vm vm)` Releases BW used by a VM. |
    | `abstract long` | `getAllocatedBwForVm(Vm vm)` Gets the allocated BW for VM. |
    | `long` | `getAvailableBw()` Gets the available BW in the host. |
    | `long` | `getBw()` Gets the bw. |
    | `long` | `getUsedBw()` Gets the amount of used BW in the host. |
    | `abstract boolean` | `isSuitableForVm(Vm vm, long bw)` Checks if BW is suitable for vm. |
    | `protected void` | `setAvailableBw(long availableBw)` Sets the available bw. |
    | `protected void` | `setBw(long bw)` Sets the bw. |

    - ### Methods inherited from class java.lang.Object

      `clone, equals, finalize, getClass, hashCode, notify, notifyAll, toString, wait, wait, wait`

- - ### Constructor Detail


    - #### BwProvisioner

      ```
      public BwProvisioner(long bw)
      ```

      Creates the new BwProvisioner.

      Parameters:
      :   `bw` - overall amount of bandwidth available in the host.
  - ### Method Detail


    - #### allocateBwForVm

      ```
      public abstract boolean allocateBwForVm(Vm vm,
                            long bw)
      ```

      Allocates BW for a given VM.

      Parameters:
      :   `vm` - virtual machine for which the bw are being allocated
      :   `bw` - the bw

      Returns:
      :   $true if the bw could be allocated; $false otherwise


    - #### getAllocatedBwForVm

      ```
      public abstract long getAllocatedBwForVm(Vm vm)
      ```

      Gets the allocated BW for VM.

      Parameters:
      :   `vm` - the VM

      Returns:
      :   the allocated BW for vm


    - #### deallocateBwForVm

      ```
      public abstract void deallocateBwForVm(Vm vm)
      ```

      Releases BW used by a VM.

      Parameters:
      :   `vm` - the vm


    - #### deallocateBwForAllVms

      ```
      public void deallocateBwForAllVms()
      ```

      Releases BW used by a all VMs.


    - #### isSuitableForVm

      ```
      public abstract boolean isSuitableForVm(Vm vm,
                            long bw)
      ```

      Checks if BW is suitable for vm.

      Parameters:
      :   `vm` - the vm
      :   `bw` - the bw

      Returns:
      :   true, if BW is suitable for vm


    - #### getBw

      ```
      public long getBw()
      ```

      Gets the bw.

      Returns:
      :   the bw


    - #### setBw

      ```
      protected void setBw(long bw)
      ```

      Sets the bw.

      Parameters:
      :   `bw` - the new bw


    - #### getAvailableBw

      ```
      public long getAvailableBw()
      ```

      Gets the available BW in the host.

      Returns:
      :   available bw


    - #### getUsedBw

      ```
      public long getUsedBw()
      ```

      Gets the amount of used BW in the host.

      Returns:
      :   used bw


    - #### setAvailableBw

      ```
      protected void setAvailableBw(long availableBw)
      ```

      Sets the available bw.

      Parameters:
      :   `availableBw` - the new available bw


- Overview
- Package
- Class
- Use
- Tree
- Deprecated
- Index
- Help

- Prev Class
- Next Class

- Frames
- No Frames

- All Classes

- Summary:
- Nested |
- Field |
- Constr |
- Method

- Detail:
- Field |
- Constr |
- Method
